# Case report: BRAF A598-T599insV mutation as a potential resistance mechanism to alectinib in ALK-rearranged lung adenocarcinoma

**DOI:** 10.3389/fonc.2022.985446

**Published:** 2022-11-07

**Authors:** Thomas Pasau, Els Wauters, Isabelle Wauters, Fabrice Duplaquet, Lionel Pirard, Claudia Pop-Stanciu, Nicky D’Haene, Michael Dupont, Thierry Vander Borght, Benoît Rondelet, Sebahat Ocak

**Affiliations:** ^1^ Division of Pulmonology, Centre Hospitalier Universitaire de l'Université Catholique de Louvain (CHU UCL) Namur (Godinne Site), Université Catholique de Louvain, Yvoir, Belgium; ^2^ Respiratory Oncology Unit (Pulmonology), University Hospitals Katholieke Universiteit Leuven, Leuven, Belgium; ^3^ Laboratory of Respiratory Diseases and Thoracic Surgery (BREATHE), Department of Chronic Diseases and Metabolism, Katholieke Universiteit Leuven, Leuven, Belgium; ^4^ Division of Pathology, Centre Hospitalier Universitaire de l'Université Catholique de Louvain (CHU UCL) Namur (Godinne Site), Université Catholique de Louvain, Yvoir, Belgium; ^5^ Department of Pathology, Hôpital Erasme, Université Libre de Bruxelles, Brussels, Belgium; ^6^ Division of Radiology, Centre Hospitalier Universitaire de l'Université Catholique de Louvain (CHU UCL) Namur (Godinne Site), Université Catholique de Louvain, Yvoir, Belgium; ^7^ Division of Nuclear Medicine, Centre Hospitalier Universitaire de l'Université Catholique de Louvain (CHU UCL) Namur (Godinne Site), Université Catholique de Louvain, Yvoir, Belgium; ^8^ Division of Thoracic Surgery, Centre Hospitalier Universitaire de l'Université Catholique de Louvain (CHU UCL) Namur (Godinne Site), Université Catholique de Louvain, Yvoir, Belgium; ^9^ Pole of Pulmonology, Institut de Recherche Expérimentale et Clinique, Université Catholique de Louvain, Brussels, Belgium

**Keywords:** *ALK* rearrangement, *BRAF* A598-T599insV mutation, lung adenocarcinoma, resistance to alectinib, non-small cell lung cancer

## Abstract

Anaplastic lymphoma kinase (ALK) tyrosine kinase inhibitors (TKIs) have improved the prognosis of advanced-stage non-small cell lung cancer (NSCLC) with ALK rearrangement, but resistance mechanisms limit their efficacy. We describe the case of a 63-year-old man with a stage cIVA *ALK*-rearranged lung adenocarcinoma who developed a *BRAF* A598-T599insV mutation as a potential resistance mechanism to alectinib, a second-generation ALK TKI. He was treated with an association of BRAF and MEK inhibitors but death occurred two months after treatment initiation in a context of tumor progression and toxicity. Based on this first report of *BRAF* A598-T599insV mutation occurring in lung cancer, we discuss resistance mechanisms to ALK TKIs, implications of *BRAF* mutation in NSCLC, and *BRAF* A598-T599insV mutation in other cancers.

## Introduction

Lung cancer is the leading cause of cancer-related mortality worldwide, responsible for 1.5 million deaths per year (1). There are two histological types: non-small cell lung cancer (NSCLC) and small cell lung cancer (SCLC), representing ≃85% and ≃15% of cases respectively (2). NSCLC is further divided into three histological subtypes: adenocarcinoma, squamous cell carcinoma, and large cell carcinoma, representing ≃40-50%, ≃30%, and ≃10% of cases respectively. In NSCLC, oncogenic drivers such as *EGFR*, *BRAF* V600E, *MET* exon14, *KRAS* G12C mutations, and Anaplastic lymphoma kinase (*ALK)*, *ROS*-1, *RET*, and *NTRK* rearrangements have been identified and led to personalized medicine after clinical trials showed that targeted therapies against these abnormalities improved outcomes as compared to chemotherapy (3, 4).

*ALK* rearrangement was discovered in NSCLC in 2007. It results from an interchromosomal inversion within chromosome 2’s short arm, leading to *ALK*’s 3’ end fusion with Echinoderm microtubule-associated protein-like 4 (*EML*4)’s 5’ end or, less frequently, another gene (e.g.: *KIF5B*, *HIP1*, *TPR*, *BIRC6*). The resulting protein is activated and leads to cancer development through the activation of downstream signaling pathways such as the mitogen-activated protein kinase (MAPK), phosphatidylinositol 3-kinase (PI3K)/protein kinase B (AKT), and Janus kinase (JAK)/signal transducer and activator of transcription (STAT) pathways. *ALK* rearrangement, observed in 2-7% of NSCLCs, is more frequent in patients with adenocarcinoma, never/light-smoking history, and younger age (5, 6).

Crizotinib was the first ALK tyrosine kinase inhibitor (TKI) evaluated in *ALK*-rearranged NSCLC. Randomized trials showed that overall response rate (ORR) and progression-free survival (PFS) were better with crizotinib than chemotherapy in first- and further-line treatment of advanced-stage *ALK*-rearranged NSCLC. However, resistance mechanisms occur inevitably, responsible for tumor progression. Second-generation (alectinib, ceritinib, brigatinib, and ensartinib) and third-generation (lorlatinib) ALK TKIs have therefore been developed, but also face resistance issues. ALK-dependent resistance mechanisms consist mainly of mutations in *ALK* tyrosine kinase domain (altering kinase conformation and/or ATP binding affinity and preventing TKI binding) and less frequently of *ALK* amplification. ALK-independent mechanisms include bypass and downstream signaling activation (6–11).

In this report, we present a *BRAF* A598-T599insV mutation as a new potential ALK-independent resistance mechanism to alectinib in a patient with metastatic *ALK*-rearranged lung adenocarcinoma. We also discuss literature related to resistance to ALK TKIs, *BRAF* mutation in NSCLC, and *BRAF* A598-T599insV mutation in other cancers.

## Case presentation

**Figure 1:**
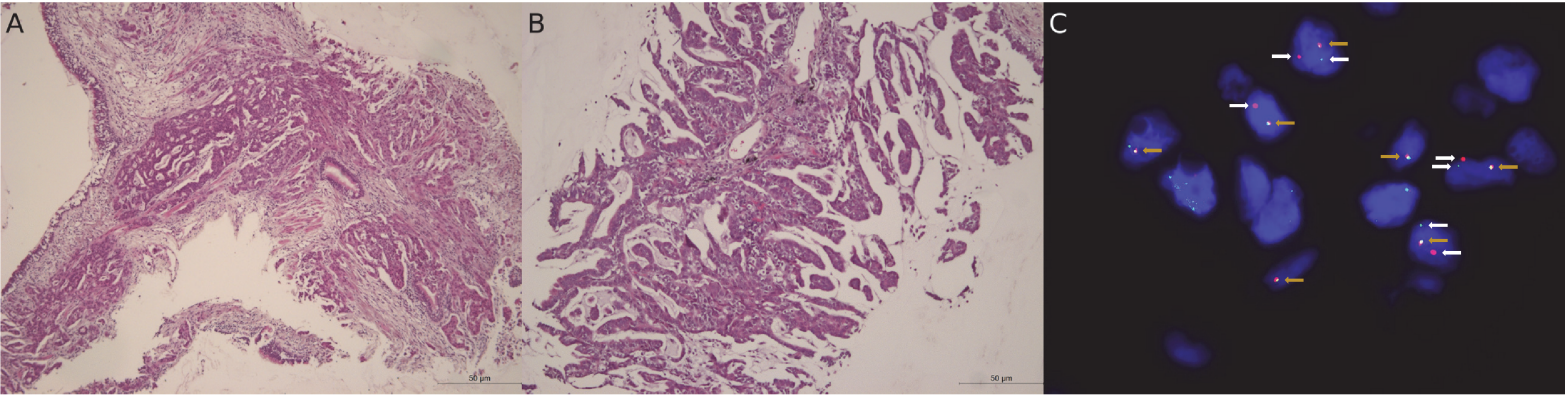
Pathological and molecular analysis of tumor samples. **(A, B)**. Hematoxylin and eosin (HE) staining shows neoplastic cells with morphological characteristics of lung adenocarcinoma. **(A)** *Picture magnification: 5x; scale bar: 50 µm.* **(B)** *Picture magnification: 10x; scale bar: 50 µm.* **(C)** Fluorescent *in situ* hybridization (FISH) reveals an *ALK* rearrangement (IQFISH break apart DAKO (Omnis)). ALK break-apart FISH utilizes DNA probes that hybridize to the 3’ (red signal) and 5’ (green signal) regions of the common fusion breakpoint in ALK gene. *ALK* rearrangement is identified by splitting of the red and green signals in the nuclei (white arrows) or isolated red signals, as opposed to fused adjacent red and green signals (yellow arrows). *Picture magnification: 1000x*.

In 2016, a 63-year-old never-smoking male patient, with a history of resected prostatic adenocarcinoma in 2006, was diagnosed with a left lower lobe lung adenocarcinoma, stage cIVA (UICC 7^th^ edition) (cT2a cN3 cM1a (metastases in the contralateral lung)). While there was no oncogenic driver found by a DNA Next Generation Sequencing (NGS) panel (Ion Torrent, ThermoFisher) targeting 22 genes (**Supplementary Table 1**), further molecular analyses revealed an *ALK* rearrangement (score 2+ ALK expression by immunohistochemistry, confirmation by fluorescent *in situ* hybridization (FISH) (38% of analyzed tumor cells were positive) and by RNA NGS (11.115 reads), which revealed an Echinoderm microtubule-associated protein-like 4 (*EML4*) (12)-*ALK* (12) fusion) (**Figure 1**).

**Figure 2:**
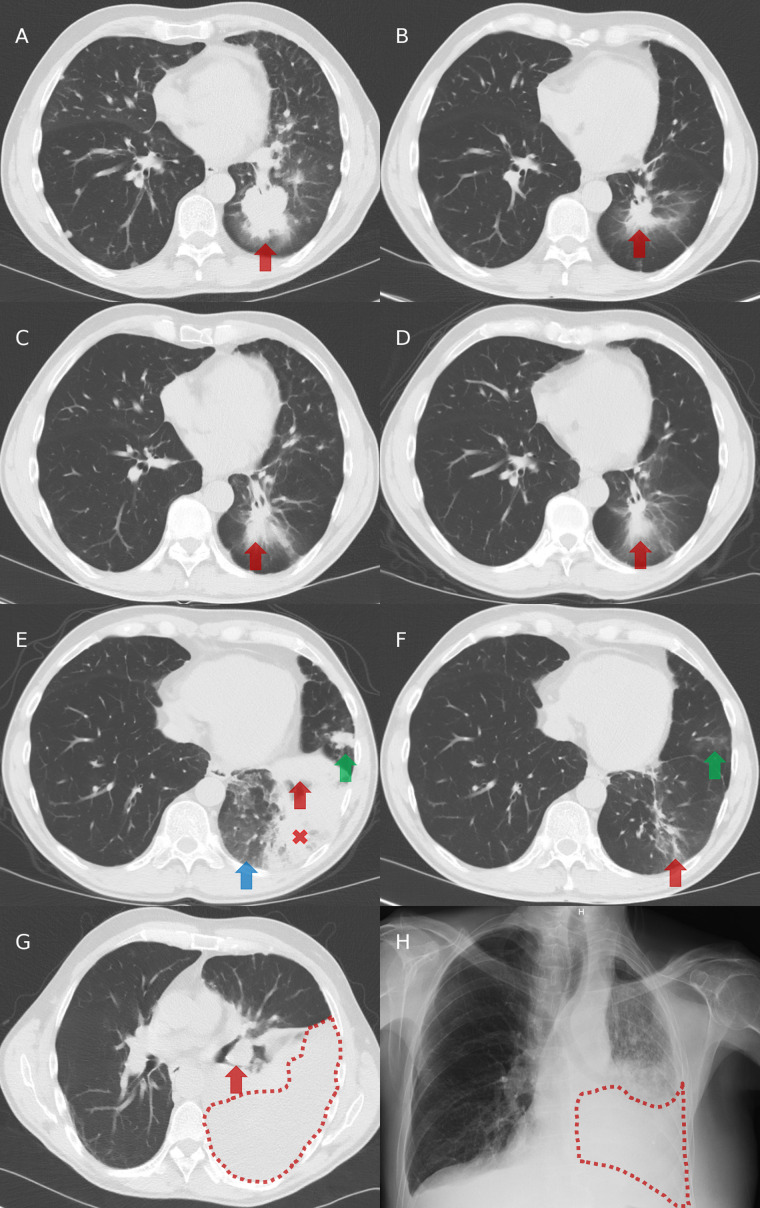
Chest imaging evolution. **(A)** Computed tomography (CT) at diagnosis: presence of a left-lower lobe primary tumor (red arrow). **(B)** CT after three cycles of cisplatin and pemetrexed in first-line: partial tumor response with decreased size of the primary tumor (red arrow). **(C)** CT after 7 cycles of pemetrexed in maintenance and then 2.5 months without systemic treatment: tumor progression with increased size of the primary tumor (red arrow). **(D)** CT after two months on crizotinib in second-line: partial tumor response with decreased size of the primary tumor (red arrow). **(E)** CT after 5.5 months on crizotinib in second-line: progression of the primary tumor (red arrow), appearance of ground glass opacities (blue arrow), a retro-obstructive condensation in the left lower lobe (red cross), and a nodular lesion in the lingula (green arrow). **(F)** CT after two months on alectinib in third-line: partial tumor response with decreased size of the left lower lobe primary tumor (red arrow) and of the lingular nodule (green arrow). **(G)** CT after 15 months on alectinib: tumor progression with increased size of the primary tumor (red arrow) and appearance of a left pleural effusion (area under red dots). **(H)** Radiography after two months on BRAF/MEK inhibitors in fourth-line: left pleural effusion (area under red dots).

In 2016 in Belgium, ALK TKIs were not reimbursed in first-line and so, the patient initially received cisplatin-pemetrexed chemotherapy, with partial tumor response observed after three (**Figures 2A, B**) and five cycles. Then, pemetrexed maintenance was initiated but stopped after seven cycles despite stable disease because of a grade 2 asthenia and a grade 1 renal failure. He experienced tumor progression 2.5 months after stopping pemetrexed (**Figure 2C**).

Crizotinib was initiated in second-line, with partial response achieved after two months (**Figure 2D**) and disease progression observed in the left lung after 5.5 months (**Figure 2E**). Tumor re-biopsies showed persistence of an *ALK* rearrangement (30% of analyzed tumor cells positive by FISH and 15.431 reads by RNA NGS) but no resistance mechanism to crizotinib (screening with a DNA NGS panel (Ion Torrent, ThermoFisher) targeting 22 genes, including *ALK* exons 22, 23, and 25).

In third-line, the patient received alectinib. Partial response was observed after two months (**Figure 2F**), followed by stabilization until tumor progression after 15 months (increase of the lesions in the left lower lobe and occurrence of a left pleural carcinomatosis) (**Figure 2G**). Tumor re-biopsies revealed persistence of an *ALK* rearrangement (24% of analyzed tumor cells positive by FISH and 16.286 reads by RNA NGS) and detected a *BRAF* A598_T599insV mutation (allelic frequency of 11%, screening with a DNA NGS panel (Ion Torrent, ThermoFisher) targeting 25 genes [the same 22 genes than in the panels used at diagnosis and at progression on crizotinib, plus three other genes (**Supplementary Table 2**)], while there was no *ALK* mutation.

**Figure 3:**
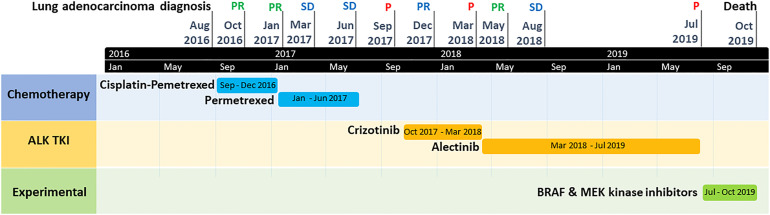
Timeline graph of patient clinical history, with tumor evolution and treatments. P, tumor progression; PR, tumor partial response; SD, tumor stable disease; TKI, tyrosine kinase inhibitor.

Therefore, we did not propose a third-generation ALK TKI in fourth-line but an experimental treatment associating BRAF and MEK kinase inhibitors. Unfortunately, the patient died two months later in a context of tumor progression (**Figure 2H**) and toxicity (grade 3 skin rash and amylase elevation). The timeline of patient clinical history, with tumor evolution and treatments, is represented in **Figure 3**.

## Discussion

We report here the *BRAF* A598_T599insV mutation as a potential new resistance mechanism to alectinib in metastatic *ALK*-rearranged lung adenocarcinoma.

After platinum-based chemotherapy, the patient was treated with crizotinib. Progression under crizotinib was observed after only 5.5 months and re-biopsies did not reveal any molecular resistance mechanism. Resistance to crizotinib is usually acquired (93-95%) and secondary to ALK-dependent or, more frequently, ALK-independent mechanisms (2/3 cases) (7). ALK-dependent resistance mechanisms include *ALK* mutations (the most frequent ones being L1196M (7%) and G1269A (4%), the less frequent ones C1156Y/T, L1152P/R, I1151Tins, F1174C/L/V, G1128A), *ALK* amplification (7-18%), and loss of *ALK* rearrangement (8, 11). ALK-independent resistance mechanisms include activation of bypass signaling pathways (e.g.: *EGFR* mutation and/or amplification, *KRAS* mutation, *KIT* amplification, *IGF-1R* activation, *RAS/MEK* activation), histological transformation to SCLC, and epithelial to mesenchymal transition (EMT) (11).

To overcome these resistance mechanisms to crizotinib, second-generation ALK TKIs have been developed. Randomized trials demonstrated their superiority over chemotherapy after progression on crizotinib, even in the absence of *ALK* mutations, which occur only in a minority of cases (~20%) in this setting (7). Patients progressing on crizotinib generally remain ALK-dependent despite the absence of ALK mutations probably because of the low potency of crizotinib against ALK. However, it is possible that sequencing panels do not adequately capture low frequency variants or previously undescribed ALK resistance mutations. In this context of tumor progression on crizotinib and absence of a specific resistance mechanism, our patient was treated with second-generation alectinib. Progression was observed after 15 months. ALK-dependent resistance mechanisms are more frequent with second-generation (1/2 cases) than first-generation TKIs and seem to increase with each successive generation of ALK TKI. The most frequent *ALK* mutation of resistance to second-generation TKIs is G1202R (21% post-ceritinib, 29% post-alectinib, and 43% post-brigatinib). Less frequent secondary *ALK* mutations are F1174C/L (17%), C1156Y (8%), G1202del (8%) post-ceritinib, I1171T/S (12%), V1180L (6%), and L1196M (6%) post-alectinib, and E1210K (29%), D1203N (14%), S1206Y/C (14%) post-brigatinib (11). ALK-independent resistance mechanisms to second-generation ALK TKIs include alterations in bypass activating pathways (such as *RET* fusion, *MET* amplification, mutation, or rearrangement, *PIK3CA*, *FGRFR2*, *MEK*, and *NRAS* mutations), SCLC transformation, and EMT (11). Similarly, third-generation TKI lorlatinib has been developed to overcome these resistance mechanisms to second-generation ALK TKIs. In a phase 2 trial with lorlatinib, analysis of plasma and tissues from 198 *ALK*-rearranged NSCLC patients showed that those with *ALK* mutations post-second-generation TKI had higher ORR than those without (62% vs 32% in plasma and 69% vs 27% in tissue) (13). While ALK-independent resistance mechanisms remain sensitive to ALK inhibition post-crizotinib due to crizotinib’s lower potency, this is no longer the case post-second/third-generation TKI’s (11).

In our patient, in the absence of an *ALK* mutation but presence of a *BRAF* A598_T599insV mutation at progression on alectinib, we proposed participation in a phase 1b clinical trial evaluating a BRAF and a MEK inhibitor in BRAF- and KRAS-mutated NSCLC and NRAS-mutated melanoma instead of a treatment with lorlatinib. *BRAF* mutations are found in 1.5-3.5% of NSCLCs at diagnosis, almost exclusively in adenocarcinoma, and are responsible for the MAPK/ERK pathway activation leading to tumor development and progression (14). Half of *BRAF*-mutated NSCLCs have a *BRAF* V600E mutation, which is more frequent in light/never-smokers and, at diagnosis, is mutually exclusive with other oncogenic drivers such as ALK rearrangement. *BRAF* V600E-mutated NSCLC’s treatment consists of a BRAF inhibitor (vemurafenib or dabrafenib) in association with a MEK inhibitor (trametinib). The response to BRAF/MEK inhibitors in presence of *BRAF* non-V600E mutations is less consistent, some of them being sensitive to BRAF/MEK inhibitors (e.g.: *BRAF* L597 and K601 mutations), while others not (e.g.: *BRAF* G464 and G469 mutations) (15). *BRAF* mutations can also occur as an acquired resistance to other targeted therapies. and have only recently been reported as a resistance mechanism to ALK TKIs. *BRAF* G15V mutation was first observed in one among 27 NSCLC patients progressing on second-generation ALK TKI (3.7%) (7). *BRAF* mutations were then reported in circulating tumor cells of 3/14 patients progressing on crizotinib (D587A in one, E586K and I592M in a second, and E586K in a third patient) (10). *BRAF* V600E mutation was observed in a patient with *ALK*-rearranged lung adenocarcinoma previously treated with crizotinib and pemetrexed (16). Because BRAF and/or MEK inhibitors were not available, the patient was treated with alectinib, but experienced tumor progression after three months. At time of progression on alectinib, rebiopsies showed persistence of the *BRAF* V600E mutation. *BRAF* V600E mutation was also found, with an *ALK* I1171T mutation, in a patient with *ALK*-rearranged lung adenocarcinoma progressing on alectinib after crizotinib (17). The patient died three months after lorlatinib was initiated. Finally, *BRAF* V600 mutation was found in a patient-derived xenograft (PDX) model from a patient progressing on alectinib (18). Triple combination of alectinib, dabrafenib, and trametinib effectively and safely suppressed tumor growth in this PDX model. To the best of our knowledge, the *BRAF* A598_T599insV mutation has never been described in lung cancer before, while only once in papillary thyroid carcinoma (19) and twice in melanoma (12, 20). Due to the rarity of non-V600E *BRAF* mutations in cancer, their clinical significance remains to be established. In this rare subset of non-V600E *BRAF* mutations, the *BRAF* A598-T599insV mutation is even rarer and its importance in cancer is unknown. In melanoma, response to BRAF/MEK inhibitors has been reported, one transient (12) and the other one complete and durable (20), suggesting that this *BRAF* A598_T599insV has an oncogenic role such as the *BRAF* V600E mutation through the activation of the MAPK/ERK pathway. In our patient, we hypothesized that the *BRAF* A598_T599insV mutation was a resistance mechanism to alectinib because it was not present at diagnosis and at progression on crizotinib but appeared at progression on alectinib. We therefore treated our patient, after stopping alectinib, with BRAF and MEK inhibitors instead of lorlatinib. However, the patient died only two months after this treatment’s initiation. Failure of the BRAF/MEK inhibitors may be explained by the fact that the *ALK* rearrangement was still present in the tumor biopsies obtained at progression on alectinib, in addition to the *BRAF* A598-T599insVmutation, these two genetic abnormalities activating different signaling pathways leading to tumor progression. Therefore, as BRAF and MEK inhibitors do not inhibit ALK and all its downstream signaling pathways, cells with *ALK* rearrangement were probably not controlled by the BRAF/MEK inhibitors targeting only the *BRAF*-mutated cells. This is often an issue in the context of acquired resistance mechanisms to oncogenic drivers, encouraging the association of multiple targeted therapies, as previously reported for instance in presence of an *EGFR* mutation and a *BRAF* V600 mutation as resistance mechanism to the third-generation EGFR TKI osimertinib (21). Even though this kind of association is interesting from a theoretical point of view, toxicity may be a problem and is the reason why we did not consider it in our patient and preferred to propose him participation in a clinical trial. In the absence of response to BRAF/MEK inhibitors, it is difficult to confirm that this *BRAF* A598-T599insV mutation was a resistance mechanism to alectinib in our patient. It is indeed possible that the *BRAF* mutation was the selection of a preexisting BRAF-clone by alectinib, even though not identified before.

## Conclusion

In a patient with *ALK*-rearranged lung adenocarcinoma progressing on alectinib, we detected a *BRAF* A598-T599insV mutation, suggesting it as a resistance mechanism to alectinib. To our best knowledge, the *BRAF* A598-T599insV mutation has never been described before in lung cancer. Further research is needed to determine whether this mutation is a resistance mechanism to alectinib in lung cancer and what is the best treatment in this setting.

## Data availability statement

The datasets for this article are not publicly available due to concerns regarding participant/patient anonymity. Requests to access the datasets should be directed to the corresponding author.

## Ethics statement

This study was reviewed and approved by Ethical Committee of CHU UCL Namur (Godinne Site). Written informed consent was not provided because the patient deceased before the writing of the manuscript and the family has not been contacted to avoid painful memories. Ethical Committee of CHU UCL Namur provided the authorization to publish this case report without written informed consent.

## Author contributions

SO made the conceptualization and supervision of the article. TP and SO wrote the original draft. EW, IW, FD, LP, CP-S, ND’H, TVB and BR reviewed the articles and gave correction. All authors contributed to the article and approved the submitted version.

## Funding

This research did not receive any specific grant from funding agencies in the public, commercial, or not-for-profit sectors.

## Conflict of interest

The authors declare that the research was conducted in the absence of any commercial or financial relationships that could be construed as a potential conflict of interest.

## Publisher’s note

All claims expressed in this article are solely those of the authors and do not necessarily represent those of their affiliated organizations, or those of the publisher, the editors and the reviewers. Any product that may be evaluated in this article, or claim that may be made by its manufacturer, is not guaranteed or endorsed by the publisher.

## Abbreviations

AKT, protein kinase B; ALK, anaplastic lymphoma kinase; BIRC6, Baculoviral IAP Repeat Containing 6; BRAF, v-raf murine sarcoma viral oncogene homolog B1; EGFR, epidermal growth factor receptor; EML4, echinoderm microtubule-associated protein-like; FISH, fluorescent *in situ* hybridization; HIP1, Huntingtin Interacting Protein 1; JAK , Janus kinase; KIF5B, Kinesin Family Member 5B; KRAS, Kirsten rat sarcoma viral oncogene homolog; MAPK, mitogen-activated protein kinase; MEK, Mitogen-Activated Protein Kinase Kinase 1; MET, hepatocyte growth factor receptor; NGS, Next Generation Sequencing; NSCLC, non-small cell lung cancer; NTRK, neurotrophic receptor tyrosine kinase; ORR, overall response rate; PFS, progression-free survival; PI3K, phosphatidylinositol 3-kinase; RET, rearranged during transfection; ROS-1, c-Ros oncogene 1; SCLC, small cell lung cancer; STAT, signal transducer and activator of transcription; TKI, tyrosine kinase inhibitor; TPR, Translocated Promoter Region, Nuclear Basket Protein.

## References

[1] Sung H Ferlay J Siegel RL Laversanne M Soerjomataram I Jemal A. Global cancer statistics 2020: GLOBOCAN estimates of incidence and mortality worldwide for 36 cancers in 185 countries. CA Cancer J Clin (2021) 71(3):209–49. doi: 10.3322/caac.21660 33538338

[2] Houston KA Henley SJ Li J White MC Richards TB. Patterns in lung cancer incidence rates and trends by histologic type in the united states, 2004-2009. Lung Cancer (2014) 86(1):22–8. doi: 10.1016/j.lungcan.2014.08.001 PMC5823254 25172266

[3] Barlesi F Mazieres J Merlio JP Debieuvre D Mosser J Lena H. Routine molecular profiling of patients with advanced non-small-cell lung cancer: Results of a 1-year nationwide programme of the French cooperative thoracic intergroup (IFCT). Lancet (2016) 387(10026):1415–26. doi: 10.1016/S0140-6736(16)00004-0 26777916

[4] Couraud S Souquet PJ Paris C Dô P Doubre H Pichon E. BioCAST/IFCT-1002: Epidemiological and molecular features of lung cancer in never-smokers. Eur Respir J (2015) 45(5):1403–14. doi: 10.1183/09031936.00097214 25657019

[5] Camidge DR Doebele RC. Treating ALK-positive lung cancer–early successes and future challenges. Nat Rev Clin Oncol (2012) 9(5):268–77. doi: 10.1038/nrclinonc.2012.43 PMC4142046 22473102

[6] Peng L Zhu L Sun Y Stebbing J Selvaggi G Zhang Y. Targeting ALK rearrangements in NSCLC: Current state of the art. Front Oncol (2022) 12:863461. doi: 10.3389/fonc.2022.863461 35463328 PMC9020874

[7] Gainor JF Dardaei L Yoda S Friboulet L Leshchiner I Katayama R. Molecular mechanisms of resistance to first- and second-generation ALK inhibitors in ALK-rearranged lung cancer. Cancer Discovery (2016) 6(10):1118–33. doi: 10.1158/2159-8290.CD-16-0596 PMC5050111 27432227

[8] Isozaki H Takigawa N Kiura K. Mechanisms of acquired resistance to ALK inhibitors and the rationale for treating ALK-positive lung cancer. Cancers (Basel) (2015) 7(2):763–83. doi: 10.3390/cancers7020763 PMC4491683 25941796

[9] Lin JJ Riely GJ Shaw AT. Targeting ALK: Precision medicine takes on drug resistance. Cancer Discovery (2017) 7(2):137–55. doi: 10.1158/2159-8290.CD-16-1123 PMC5296241 28122866

[10] Pailler E Faugeroux V Oulhen M Mezquita L Laporte M Honoré A. Acquired resistance mutations to ALK inhibitors identified by single circulating tumor cell sequencing in ALK-rearranged non-Small-Cell lung cancer. Clin Cancer Res (2019) 25(22):6671–82. doi: 10.1158/1078-0432.CCR-19-1176 31439588

[11] Tabbò F Reale ML Bironzo P Scagliotti GV. Resistance to anaplastic lymphoma kinase inhibitors: Knowing the enemy is half the battle won. Transl Lung Cancer Res (2020) 9(6):2545–56. doi: 10.21037/tlcr-20-372 PMC7815358 33489817

[12] Rogiers A Vander Borght S Tuand K Wolter P Stas M Boecxstaens V. Response to targeted therapy in two patients with metastatic melanoma carrying rare BRAF exon 15 mutations: A598_T599insV and V600_K601delinsE. Melanoma Res (2017) 27(5):507–10. doi: 10.1097/CMR.0000000000000376 28800030

[13] Solomon BJ Besse B Bauer TM Felip E Soo RA Camidge DR. Lorlatinib in patients with ALK-positive non-small-cell lung cancer: Results from a global phase 2 study. Lancet Oncol (2018) 19(12):1654–67. doi: 10.1016/S1470-2045(18)30649-1 30413378

[14] Zhang L Zheng L Yang Q Sun J. The evolution of BRAF activation in non-Small-Cell lung cancer. Front Oncol (2022) 12:882940. doi: 10.3389/fonc.2022.882940 35912223 PMC9326470

[15] Dagogo-Jack I Martinez P Yeap BY Ambrogio C Ferris LA Lydon C. Impact of BRAF mutation class on disease characteristics and clinical outcomes in BRAF-mutant lung cancer. Clin Cancer Res (2019) 25(1):158–65. doi: 10.1158/1078-0432.CCR-18-2062 30224342

[16] Urbanska EM Sørensen JB Melchior LC Costa JC Santoni-Rugiu E. Changing ALK-TKI-Resistance mechanisms in rebiopsies of ALK-rearranged NSCLC: ALK- and BRAF-mutations followed by epithelial-mesenchymal transition. Int J Mol Sci (2020) 21(8):2847. doi: 10.3390/ijms21082847 PMC7215933 32325863

[17] Sui A Song H Li Y Guo L Wang K Yuan M. BRAF V600E mutation as a novel mechanism of acquired resistance to ALK inhibition in ALK-rearranged lung adenocarcinoma: A case report. Med (Baltimore) (2021) 100(8):e24917. doi: 10.1097/MD.0000000000024917 PMC7909161 33663128

[18] Shi R Filho SNM Li M Fares A Weiss J Pham NA. BRAF V600E mutation and MET amplification as resistance pathways of the second-generation anaplastic lymphoma kinase (ALK) inhibitor alectinib in lung cancer. Lung Cancer (2020) 146:78–85. doi: 10.1016/j.lungcan.2020.05.018 32521388

[19] Torregrossa L Viola D Sensi E Giordano M Piaggi P Romei C. Papillary thyroid carcinoma with rare exon 15 BRAF mutation has indolent behavior: A single-institution experience. J Clin Endocrinol Metab (2016) 101(11):4413–20. doi: 10.1210/jc.2016-1775 27571181

[20] Bjursten S Vannas C Filges S Puls F Pandita A Fagman H. Response to BRAF/MEK inhibition in A598_T599insV BRAF mutated melanoma. Case Rep Oncol (2019) 12(3):872–9. doi: 10.1159/000504291 PMC6902217 31824282

[21] Aboubakar Nana F Ocak S. Targeting BRAF activation as acquired resistance mechanism to EGFR tyrosine kinase inhibitors in EGFR-mutant non-Small-Cell lung cancer. Pharmaceutics (2021) 13(9):1478. doi: 10.3390/pharmaceutics13091478 34575554 PMC8471192

