# Supplementary material for: Case report: BRAF A598-T599insV mutation as a potential resistance mechanism to alectinib in ALK-rearranged lung adenocarcinoma
Source: Front Oncol. 2022 Nov 7;12:985446. doi: 10.3389/fonc.2022.985446 (PMC9677532; doi:10.3389/fonc.2022.985446)
Supplement: Supplementary file 1 [file DataSheet_1.docx]

**Supplementary tables**

Supplementary table 1: DNA Next Generation Sequencing panel used to analyze biopsies obtained at diagnosis and progression on crizotinib

| **Gene** | **RefSeq** | **Tested exons** | **Non contributive exons (coverage <250x)** |
| --- | --- | --- | --- |
| AKT1 | NM_05163 | 3 |  |
| ALK | NM_004304 | 22, 23, 25 |  |
| BRAF | NM_004333 | 11,  15 |  |
| CTNNB1 | NM_001904 | 3 |  |
| DDR2 | NM_001014796 | 6, 9, 13-16, 18 |  |
| EGFR | NM_005228 | 12, 18-21 |  |
| ERBB2 | NM_004448 | 19-21 |  |
| ERBB4 | NM_005235 | 3, 4, 6-9, 15,  23 |  |
| FBXW7 | NM_033632 | 5, 8-11 |  |
| FGFR1 | NM_023110 | 4, 7 |  |
| FGFR2 | NM_022970 | 7, 9, 12 |  |
| FGFR3 | NM_000142 | 7, 9, 14, 16, 18 |  |
| KRAS | NM_033360 | 2-4 |  |
| MAP2K1 | NM_002755 | 2 |  |
| MET | NM_001127500 | 2, 14, 16, 19 |  |
| NOTCH1 | NM_017617 | 26, 27 |  |
| NRAS | NM_002524 | 2, 3, 4 |  |
| PIK3CA | NM_006218 | 9, 13, 20 |  |
| PTEN | NM_000314 | 1, 3, 6-8 |  |
| SMAD4 | NM_005359 | 3, 5, 6, 8, 9, 10, 12 |  |
| STK11 | NM_000455 | 1, 4-6, 8 |  |
| TP53 | NM_000546 | 2, 4-8, 10 |  |

Supplementary table 2: DNA Next Generation Sequencing panel used to analyze biopsies obtained at progression on alectinib

| **Gene** | **RefSeq** | **Tested exons** | **Non contributive exons (coverage <250x)** |
| --- | --- | --- | --- |
| AKT1 | NM_05163 | 3 |  |
| ALK | NM_004304 | 22, 23, 24, 25 |  |
| BRAF | NM_004333 | 11,  15 |  |
| CTNNB1 | NM_001904 | 3 |  |
| DDR2 | NM_001014796 | 6, 9, 13-16, 18 |  |
| EGFR | NM_005228 | 12, 18-21 |  |
| ERBB2 | NM_004448 | 19-21 |  |
| ERBB4 | NM_005235 | 3, 4, 6-10, 12, 15, 23 |  |
| FBXW7 | NM_033632 | 5, 8-11 |  |
| FGFR1 | NM_023110 | 4, 7 |  |
| FGFR2 | NM_022970 | 7, 9, 12, 14 |  |
| FGFR3 | NM_000142 | 7, 9, 14, 16, 18 |  |
| HRAS | NM_005343 | 2, 3, 4 |  |
| KIT | NM_000222 | 8, 9, 11, 13, 14, 17, 18 |  |
| KRAS | NM_033360 | 2-4 |  |
| MAP2K1 | NM_002755 | 2 |  |
| MET | NM_001127500 | 2, 14-20 |  |
| NOTCH1 | NM_017617 | 26, 27 |  |
| NRAS | NM_002524 | 2, 3, 4 |  |
| PDGFRA | NM_006206 | 12, 14, 18 |  |
| PIK3CA | NM_006218 | 9, 13, 20 |  |
| PTEN | NM_000314 | 1, 3, 6-8 |  |
| SMAD4 | NM_005359 | 3, 5, 6, 8-10, 12 |  |
| STK11 | NM_000455 | 1, 4-6, 8 |  |
| TP53 | NM_000546 | 2, 4-8, 10 |  |
